# Effect of Dietary Rosemary and Ginger Essential Oils on the Growth Performance, Feed Utilization, Meat Nutritive Value, Blood Biochemicals, and Redox Status of Growing NZW Rabbits

**DOI:** 10.3390/ani12030375

**Published:** 2022-02-03

**Authors:** Mahmoud A. Elazab, Ayman M. Khalifah, Abdelmotaleb A. Elokil, Alaa E. Elkomy, Marwa M. Rabie, Abdallah Tageldein Mansour, Sabrin Abdelrahman Morshedy

**Affiliations:** 1 Livestock Research Department, Arid Lands Cultivation Research Institute, City of Scientific Research and Technological Applications, Alexandria 21934, Egypt; melazab@srtacity.sci.eg (M.A.E.); akhalifah@srtacity.sci.eg (A.M.K.); alaa_elkomy@yahoo.com (A.E.E.); 2 Department of Animal Production, Faculty of Agriculture, Benha University, Moshtohor 13736, Egypt; abdelmotaleb@fagr.bu.edu.eg; 3 Faculty of Desert and Environmental Agriculture, Matrouh University, Matrouh 51512, Egypt; 4 Department of Poultry Production, Faculty of Agriculture, Mansoura University, Mansoura 35516, Egypt; m_rabie2009@mans.edu.eg; 5 Animal and Fish Production Department, College of Agricultural and Food Sciences, King Faisal University, Al-Ahsa 31982, Saudi Arabia; amansour@kfu.edu.sa; 6 Fish and Animal Production Department, Faculty of Agriculture (Saba Basha), Alexandria University, Alexandria 21531, Egypt

**Keywords:** rabbit meat quality, rosemary essential oil, ginger essential oil, growth performance, lipid profile, antioxidant balance

## Abstract

**Simple Summary:**

The rabbit farming industry has gained more interest due to its high productivity, high growth rate, and high-quality meat. One of the public health concerns is that global rabbit production is expected to increase to meet the increasing demand for lean meat. In the present study, we focused on the use of phytogenic feed additives (essential oils of rosemary (REO) and ginger (GEO)) as environmentally friendly supplementation to improve rabbit growth performance, physiological status, and meat quality. The results indicated that the use of REO and GEO at a dose of 0.5% dramatically improved the growth performance and feed utilization of treated rabbits. The cholesterol level decreased significantly in rabbit plasma and meat after REO and GEO treatments. The fat content tended to decline in the muscles and the triglycerides were remarkedly reduced in the plasma of treated animals. In addition, the oxidant/antioxidant balance in the plasma could be improved with supplementation with a high dose of REO and GEO. Accordingly, the use of REO and GEO as supplementations for growing rabbits could contribute to improving the sustainable production of the rabbit industry.

**Abstract:**

This study was conducted to assess the impacts of using two essential oils, rosemary and ginger, on growing rabbits’ performance, carcass traits, meat composition, blood biochemicals, and the redox status of growing New Zealand White (NZW) rabbits. A total of 120 unsexed NZW rabbits, 42-days-old, were assigned randomly to five experimental groups (*n* = 24, 6 replicates with 4 rabbits each). The first group received a basal diet (control), the second to fifth groups were dietary supplemented daily with rosemary essential oil (REO) and ginger essential oil (GEO) at doses of 0.25 and 0.5% for each supplementation (REO-0.25, REO-0.5, GEO-0.25, and GEO-0.5), respectively. The growth traits were studied for 7 weeks, from the 7th to the 13th week of the rabbits’ age. The results revealed that final body weight, weight gain, and average daily gain increased significantly (*p* < 0.01) in the REO-0.5 and GEO-0.5 treatments compared to the control group. Daily feed intake decreased (*p* = 0.005) in essential oil treatments. Meanwhile, the feed conversion ratio improved significantly (*p* = 0.001) in REO and GEO at the high doses compared to the control group. The weight percentages of liver and giblets increased (*p* < 0.001) with both treatments of REO and GEO compared to the control group. The dietary supplementation with REO and GEO did not affect (*p* > 0.05) the meat composition of *Longissimus dorsi* and hind leg muscles. Meanwhile, REO and GEO supplementation significantly decreased cholesterol levels in the rabbit meat. Thiobarbituric acid reactive substance concentrations decreased by 10 and 15% in the meat of REO-0.5 and GEO-0.5 treatments, respectively, compared to the other groups. In the same trend, REO and GEO treatments induced a significant (*p* = 0.001) reduction in the plasma cholesterol concentrations and triglycerides compared to the control. The total antioxidant capacity increased by 7.60% and the malondialdehyde decreased by 11.64% in the plasma of GEO-0.5 treatment than the control. Thus, the dietary supplementation of REO and GEO have a beneficial effect in improving the productivity and meat quality of growing rabbits.

## 1. Introduction

Rabbit meat has undoubtedly been a component of human nutrition for a long time. Moreover, the world’s consumption of rabbit meat grows year on year due to its good taste, special flavor, and diverse uses in preparing a wide variety of foods [1,2]. Commonly, rabbit meat is consumed in Egypt and several Mediterranean countries. [3,4]. Furthermore, rabbit meat is especially beneficial in Western countries, where people’s diets are often high in lipids and salt, putting them at risk of obesity, cardiovascular disease, and hypertension [3].

Recently, the rabbit industry has gained much more interest due to the fact that rabbit meat has several benefits, which qualify it to become one of the most promising healthy foods [5]. Rabbit meat is an excellent source of nutrients, including proteins, B vitamins, and minerals [4]. The functional proteins in rabbit meat have been recognized as one of the highest quality proteins in digestibility, as well as amino acid composition. Furthermore, rabbit meat is free of uric acid and has a low purine level [1,2]. In addition, rabbit meats have lower contents of salt, fat, cholesterol (59 mg/100 g of muscle) and have a lower energy value (789 kJ/100 g meat) than other species’ meat [6,7]. Furthermore, the majority of their energy comes from proteins [4]. The fatty acid composition of rabbit meat is characterized by a high polyunsaturated fatty acids (PUFA) content, especially omega-3, PUFA, which plays an important function in human nutrition by assisting in the prevention of lifestyle diseases [8,9].

The different production factors, especially feeding, have a strong influence on growth performance, reproduction, and product quality [10], as well as the chemical composition of rabbit meat, particularly regarding fat content [2]. For this reason, natural sources of feed additives can be used as a significant tool in rabbit nutrition for improving growth, feed efficiency, and reproduction, as well as lowering disease incidence and the house emissions of rabbits [11,12,13]. There is a significant motivation for phytogenic feed additives as a potential alternative to using synthetic antibiotics as growth promoters since the European Union banned growth promoters in 2006. In addition, the regulations are being tightened in the United States [14,15,16]. This ban is due to safety concerns about bacterial resistance to the synthesized antibiotics and the hazardous residuals in meat, milk, and eggs, which would pose a great threat to human health [17,18].

In this regard, probiotics, organic acids, exogenous enzymes, propolis, and plant secondary compounds, such as saponins, tannins, and essential oils (EOs) have all been recommended as natural alternatives [16]. Aromatic plants contain EOs, which can be used as phytogenic feed additives, these oils are characterized as volatile, odorous, hydrophobic, and highly concentrated compounds [19]. Essential oils are aromatic oily liquids extracted by distillation from various plant components such as flowers, buds, seeds, leaves, twigs, bark, wood, fruits, and roots [20,21,22].

Among all herbs and spices, rosemary (*Rosmarinus officinalis* L.) could be considered to contain the highest level of biologically active compounds [23]. Rosemary extracts, which are primarily made from dried rosemary leaves, are popular in feed additives and the pharmaceutical business because they have numerous health benefits, including antioxidant, antibacterial, anti-inflammatory, and anticancer properties as well as cognitive-enhancing potential [24]. The essential oil of rosemary (REO) contains several compounds at different concentrations.

Ginger from the Zingiberaceae family (*Zingiber officinale*) has long been used as a spice and herbal remedy. There are several terpene components in ginger essential oil, such as β-bisabolene, α-curcumene, zingiberene, α-farnesene, and β-sesquiphellandrene [25,26]. Ginger has pharmacological properties that manage and prevent gastrointestinal disorders, neurodegenerative diseases, atherosclerosis, cardiovascular complications, liver and kidney failure, diabetes, metabolic syndrome, cancer, and emesis/nausea [27]. In addition, ginger’s constituents participate in biological processes, such as apoptosis, DNA deterioration, chromatin and epigenetic regulation, regulation of cytoskeletal and adhesion, immunology and inflammation, and neuroscience [25,28].

Diets containing rosemary or ginger root significantly improved the growth performance of growing rabbits [3,29,30]. Moreover, feeding diets enriched with EOs significantly affected the carcass traits of rabbits [3,5]. Several studies found that EOs decreased both cholesterol and triglyceride levels [31,32,33,34]. Thus improving the oxidative stability and effectively delaying the lipid oxidation of rabbit meat by the dietary supplementation of rosemary aqueous extracts [3] or ginger powder [35].

Essential oils have been shown to improve the synthesis of digestive secretions and nutrient absorption in animals, as well as lower pathogenic stress in the gut, exert antioxidant characteristics, and strengthen the immune system, which helps to explain the observed improvement in their performance [36,37]. It is hypothesized that the dietary addition of rosemary essential oil (REO) and ginger essential oil (GEO) is expected to exert beneficial effects on growth performance, feed utilization, blood biochemicals, antioxidant status, carcass traits, and meat quality of growing rabbits. Therefore, the current study was designed to evaluate the effect of the dietary inclusion of two levels of REO and GEO (0.25 and 0.5%) in four experimental treatments on the performance, blood biochemicals, and meat quality of NZW growing rabbits.

## 2. Materials and Methods

### 2.1. Ethics Approval and Consent to Participate

Rabbits were handled in the present study following the guidelines of the Pharmaceutical & Fermentation Industries Development Center, City of Scientific Research and Technology Applications, (SRTA-City), Alexandria, Egypt, after the approval of the Institutional Animal Care and Use Committees (IACUCs)/IACUC # 37-6F-1021.

### 2.2. Animals, Experimental Design, and Housing Environment

A total of 120 unsexed NZW rabbits, aged 6 weeks, with an average body weight of 850 ± 50 g were used in this experiment. Rabbits were divided randomly into five groups (*n* = 24, 6 replicates in each group with 4 rabbits in each replicate). The 1st group received a basal diet (control). The 2nd to 5th groups were dietary supplemented daily with rosemary essential oil (REO) and ginger essential oil (GEO) at doses of 0.25 and 0.5% for each supplement (REO-0.25, REO-0.5, GEO-0.25, and GEO-0.5), respectively. The essential oils were weighed daily for each dose and added to the half amount of the ration and mixed well with pelleted basal diet to ensure the consumption of the actual dose of EOs and avoiding the loss of EOs with uneaten feed or auto-oxidation. In addition, to avoid the effect of the solvent (oil) of the essential oils, corn oil was supplemented to the basal diet of the control at a level of 0.25%. The next portion was added after the complete intake of the first portion. The experimental treatments lasted 7 weeks from the 7th to the 13th week of the rabbits’ age.

The present study was conducted at a private farm located in Borg El-Arab city, Alexandria Governorate, Egypt during February and March 2020. A total of 4 rabbits in each replicate were housed in a galvanized wire cage (dimensions: 60 cm × 40 cm × 30 cm) with a feeder and an automatic nipple drinker. All rabbits were kept under similar management, hygienic and environmental conditions throughout the experimental period. The average ambient temperature was 18–23 °C and the daily photoperiod was a 16:8 h light-dark cycle with a semi-continuous lighting program. The basal experimental diet was formulated and pelleted to meet the nutrient requirements of rabbits, according to NRC [38]. The ingredients of the basal experimental diet are shown in Table 1. The composition of the basal diet (Table 1) was calculated according to Villamide, et al. [39]. The pelleted diets and freshwater were provided ad libitium.

### 2.3. Chemical Analysis for Active Components of Rosemary and Ginger Essential Oils

The individual essential oils (EOs) of rosemary (*Rosmarinus officinalis* L.) and ginger (*Zingiber Officinalis*) were produced by the El-Hawag Factory for the extraction of Natural Oils and Cosmetics in Badr City, Egypt. The volatile contents of the oils were determined by the gas chromatography-mass spectrometry technique (GC-MS) (Table 2). The analysis of GC-MS was carried out using a Shimadzu capillary gas chromatographic system directly attached to the mass spectrometer (GC-MS–model QP 2010; (Shimadzu) DB–5 ms non-polar fused silica capillary column (30 m × 0.25 mm, 0.25 m film thickness) under the following conditions: oven temperature increased with a rate of 3 °C/min from 70 to 200 °C, and then maintained for 35 min, injection temperature: 200 °C, injection volume: 1 μL, split ratio: 100:1, carrier gas: helium, gas flow rate: 1.51 mL/min, linear velocity: 45.1 cm/s, Mass spectra were obtained at 70 eV of ionization energy, ionization source temperature: 200 °C.

### 2.4. Growth Performance Measurements

Rabbits were individually weighed in the morning before offering the feed. The initial and final body weights (BW) were recorded using a digital balance. The body weight gain (BWG) was calculated as the difference between final and initial BW and the average daily gain (ADG) was calculated as BWG divided by the number of days of the experimental period (49 days). Daily feed intake (FI) was recorded for each replicate throughout the whole experimental period, as the difference between the offered and refused feed. The feed conversion (FCR) ratio was calculated by dividing average daily feed intake/average daily gain.

### 2.5. Carcass Traits and Meat Composition

At the end of the experiment, six rabbits per treatment were randomly chosen for carcass evaluations. The rabbits were weighed pre-slaughter after fasting for 12 h then slaughtered by cutting the carotid artery and jugular vein for complete depletion. Just after bleeding, carcasses were skinned and eviscerated. The hot eviscerated carcass with a head, giblets (liver, heart, kidneys), and spleen were weighed. The carcass yields were determined as a proportion of the rabbits’ pre-slaughter live body weight. Additionally, the following equations are the percentages of total edible components, non-edible portions, and giblets:

Giblets% = kidney% + heart% + liver%.(1)

Total edible parts% = hot carcass% + Giblets%.(2)

Non-edible parts% = 100 − total edible parts%.(3)

The carcass was chilled for 24 h at 3 °C to evaluate the quality of rabbit meat. The chilled carcass was then dissected and their *Longissimus dorsi* (LD) and hind leg (HL) muscles were excised according to the recommended procedures of the World Rabbit Science Association [40]. The samples of LD and HL were individually vacuum packaged and stored at −20 °C until analyses. Meat samples were chemically analyzed for the moisture, crude protein, ether extract, and ash contents according to AOAC [41]. The cholesterol content of meat samples was determined according to the procedure described by Dinh, et al. [42]. The lipid oxidation status of LD samples was measured using the thiobarbituric acid reactive substances (TBARS) method and its products were expressed as malondialdehyde (MDA) equivalents (mg MDA/kg muscle) according to the method of Dal Bosco, et al. [43].

### 2.6. Plasma Biochemical and Antioxidant Status

Six blood samples from each treatment were obtained concurrently at slaughter in heparinized test tubes, centrifuged for separating plasma, and frozen at −20 °C for further examination. The plasma concentration of triglycerides, total cholesterol, and high-density lipoprotein-cholesterol (HDL-c) and low-density lipoprotein-cholesterol (LDL-c) were estimated colorimetrically by using commercial kits produced by (Biodiagnostic^®^ kit, Egypt). In addition, total antioxidant capacity (TAC) and MDA concentrations were measured colorimetrically according to Koracevic, et al. [44] and Banjare, et al. [45], respectively.

### 2.7. Statistical Analysis

The effect of EOs on the measured variables was analyzed statistically by one-way analysis of variance (ANOVA) using a completely randomized design. The statistical analysis was conducted using SPSS11.0 statistical software. The statistical model was used as follows:

x_ij_ = µ +T_i_ +e_ij_,

where x_ij_ is the value of the measured variable, µ is the overall mean, T_i_ is the effect of treatment (i = 5 treatments), and e_ij_ is the residual error. Duncan’s multiple range test [46] was used to compare treatment means wherever significant differences were detected at a *p*-value equal to or less than 0.05 for normally distributed data, for the non-normally distributed data, James—Howell was used as a post hoc measure for multiple comparisons. The percentage data were transformed to arc sign before the analysis [47], but the data were presented as a percentage.

## 3. Results

### 3.1. Growth Performance of Growing Rabbits

The results of growth performance of the growing NZW rabbits as affected by the dietary inclusion of REO and GEO from 7th to 13th weeks of age are shown in Table 3. The results showed that FBW, BWG, and ADG significantly increased (*p* < 0.001) in REO-0.5 and GEO-0.5 groups compared to the control group. Daily feed intake of rabbits that received REO and GEO at the highest dose were significantly lower (*p* = 0.005) than other groups. The dietary supplementation with REO and GEO at the highest doses improved (*p* = 0.001) FCR compared to the control group.

### 3.2. Carcass Traits

Table 4 shows the effect of the dietary supplementation of REO and GEO on growing NZW rabbits’ carcass traits at the end of the experiment. The dietary supplementation of REO and GEO did not affect (*p* > 0.05) the carcass characteristics of the growing NZW rabbits. The weight percentages of liver and giblets increased (*p* < 0.001) with the dietary supplementation of REO and GEO at different doses compared to the control group.

### 3.3. Meat Composition

Table 5 illustrates the effect of the dietary supplementation of REO and GEO on the composition of LD and HL muscles of the growing NZW rabbits at the end of the experiment. There were no significant differences (*p* > 0.05) among treatments on the meat composition of LD and HL muscles. Meanwhile, TBAR’s concentration tended to decrease with increasing the supplementation of REO and GEO at the highest doses compared to the other groups. The cholesterol concentration in LD muscles decreased (*p* < 0.05) with the dietary supplementation of REO and GEO at a high level compared to the control group. In the same trend, the cholesterol concentration in the HL muscles decreased (*p* < 0.05) with the dietary supplementation of REO and GEO at both levels compared to the control group.

### 3.4. Lipids Profile and Oxidant/Antioxidant Balance

**Figure 1:**
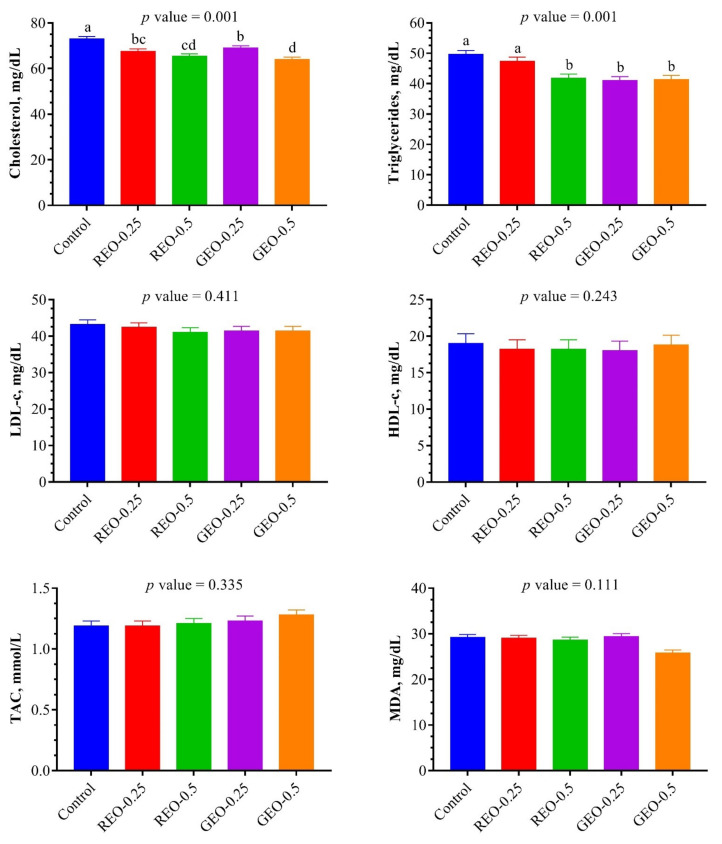
Effect of the dietary supplementation of rosemary and ginger essential oils on lipids profile and oxidant/antioxidant balance in plasma of growing NZW rabbits at the end of experiment (*n* = 6, means ± SEM). Bars bearing different superscripts are significantly different (*p* ≤ 0.05). REO-0.25 and REO-0.5: rosemary essential oil supplemented with 0.25 and 0.5%, respectively. GEO-0.25 and GEO-0.5: ginger essential oil supplemented with 0.25 and 0.5%, respectively. LDL-c: low-density lipoprotein-cholesterol; HDL-c: high-density lipoprotein-cholesterol; TAC: total antioxidant capacity; MDA: malondialdehyde.

The effect of the dietary supplementation of REO and GEO on some blood plasma parameters at the end of the experiment of the NZW rabbits from 7th to 13th weeks of age are summarized in Figure 1. Plasma cholesterol concentration was lower (*p* = 0.001) with REO or GEO treatments than in the control group. Similarly, the REO-0.5 and GEO at both doses significantly reduced (*p* = 0.001) plasma triglyceride as compared to the REO-0.25 and the control groups. However, the dietary supplementation of REO and GEO had no significant effects (*p* > 0.05) on plasma HDL-c, LDL-c levels. The dietary supplementation of GEO at the highest dose tended to improve TAC concentrations in plasma and decrease plasma MDA concentrations.

## 4. Discussion

Essential oils exhibit pharmacological properties, such as antibacterial, antimicrobial, antifungal, and antiparasitic properties, which might be due to secondary metabolites found in the oil during processing. Essential oils are low-molecular-weight chemicals that may easily permeate cell membranes and hence participate in metabolic events in the body [48].

In the present study, the dietary supplementation of REO or GEO improved the growth performance of rabbits and the FCR. These improvements could be due to the effect of essential oils on digestion, absorption, and utilization of dietary nutrients [36,37]. In addition, the effects of ginger phytochemicals are exerted by specific signaling pathways linked with the mechanisms and functions of cells, including autophagy, cellular metabolism, mitogen-activated protein kinase, and cell development and differentiation [49]. The present results are consistent with the findings of Cardinali, et al. [3], who found that dietary supplementation with rosemary (0.2%) alone or in combination with oregano extract (0.1% oregano extract + 0.1% rosemary extract) to NZW rabbits had a significant positive effect on FBW and ADG. In addition, diets containing 3% of rosemary powder or 1.5% of ginger root powder significantly improved BWG, WBG, and FCR of NZW rabbits [29]. Additionally, the increasing of ginger powder levels in growing rabbits’ diets (1, 2, and 3%) enhanced growth performance and this may be due to improving the appetite and feed utilization [30]. However, in the present study feed intake reduced with the increasing supplementation levels of both essential oils, this may be due to the high concentrations of active molecules in the essential oils in comparison to the dried ginger powder, which could cause an irritating odor and repellent smell of the ration [16]. On the other hand, the growth performance of growing rabbits was not affected by the dietary inclusion of REO at a level of 0.15% [50] or 0.25, 0.5, and 0.75 g/kg of diet [51]. Previous studies have shown that herbal oils can help in improving growth performance by increasing feed intake and/or stimulating the secretion of enzymes, resulting in better nutrient digestion and absorption through the gut [52,53]. In the present findings, there was an insignificant effect on the dietary supplementation with REO and GEO on the carcass traits of NZW rabbits. In agreement with the current results, El-Gogary, et al. [51] reported that the dietary supplementation of REO had no positive effect on carcass traits of NZW rabbits. Similarly, feeding diets enriched with peppermint essential oil, basil essential oil, or both did not significantly affect most carcass traits of rabbits [54]. However, the rabbits fed on diets supplemented with oregano extract, rosemary extract, or a combination displayed a significantly higher carcass yield (%) than the control rabbits [3]. Furthermore, nutritional supplementation with thyme essential oil enhanced carcass criteria and reduced perirenal and scapular fat without affecting rabbit internal organs [5]. However, rabbits supplemented with REO and GEO showed significantly higher liver weight percentages and giblets than the control group.

In the current results, the composition of the LD and HL muscles was not significantly affected by the dietary supplementation of REO and GEO to growing rabbits. In agreement with the present findings, diets supplemented with oregano extract, rosemary extract or a combination had no effect on the composition of the hind leg meat of growing rabbits [3]. Similarly, Hemat, et al. [55] found that feeding rabbits on diets containing remnants of mint, fennel, basil, and anise did not alter the chemical composition of the rabbitmeat.

The current findings showed a lowering in cholesterol of both the LD and HL muscles of growing rabbits due to supplementation with rosemary and ginger essential oils. Moreover, there was a significant reduction in the concentration of plasma cholesterol and triglycerides of growing rabbits treated with REO and GEO. The present results were supported by several studies that found that essential oils decreased levels of both cholesterol and triglyceride in rats due to containing limonene, which is the main active component in REO in the present study, as well as citrus EO [31,32,56,57]. Moreover, lemon EO supplementation caused an 18% decrease in triglyceride in rabbits [48]. In addition, ginger EO reduced hepatic lipid accumulation in rats [33]. Moreover, linalool (one of the active components of REO) reduced cholesterol and triglyceride levels [58]. In addition, ginger EO showed lower cholesterol and triglyceride by 21% and 24%, respectively, in male Japanese quail [34] due to the presence of zingiberene, the main constituent in GEO in the present results.

It is noteworthy that high cholesterol is a prevalent problem, as 40% of the world’s population has cholesterol levels that are above the recommended limit of 200 g/dL according to the World Health Organization, which could cause severe health problems [48,59,60]. When alternative treatments are used, the risk of side effects of therapeutic drugs for high cholesterol can be minimized [60]. Essential oils contain monoterpenoids and sesquiterpenoids such as 1,8 cineole, citral, farnesol, geraniol, limonene, and linalool, which are emerging as potential lipid-lowering agents with promising cholesterol-lowering effects [60].

In the present study, the observed reduction in cholesterol and triglyceride concentrations of rabbits treated with REO and GEO could be associated with the reduction of hepatic lipid accumulation or the inhibition of the hepatic biosynthesis of cholesterol [60]. Furthermore, the reduction of cholesterol may be due to the regulating effect of terpene derivatives in essential oils on sterol regulatory element-binding protein-1c, which lead to decreased transcription and accelerated degradation of HMG-CoA reductase (statins) as the main cholesterol synthesis pathways [33,60]. Another explanation for reducing cholesterol and triglycerides is stimulating the conversion of cholesterol to bile acids which are excreted from the body through the enterohepatic circulation [61,62]. On the other hand, insignificant effects of REO and GEO supplementation to rabbits on HDL-c, LDL-c plasma levels were found in the present study. Similar to our results, the dietary supplementation of REO did not affect plasma HDL-c or LDL-c in NZW rabbits [51].

In the present study, the supplementation with both REO and GEO at high doses decreased free radical and lipid oxidation in meat by lowering TBARS levels by 10 and 15%, respectively. In accordance with the present results, the dietary supplementation of oregano and rosemary aqueous extracts declined the TBARS concentration of the LD meat compared to the control group, thus resulting in improving the oxidative stability and effectively delaying the lipid oxidation of the LD meat of rabbits [3]. Furthermore, the dietary supplementation with ginger powder decreased the sensitivity of rabbit meat to lipid oxidation, thus offering a promising way to improve rabbit meat quality [35]. Rosemary oil exhibits effective antioxidant activity due to consisting of considerable amounts of limonene, α-pinene, camphor, and (Z)-linalool oxide [63]. Ginger oil has strong antioxidant activities because it contains active compounds including zingiberene, camphene, ar-curcumene, and b-sesquiphellandrene [64].

In muscles and fatty tissues, the oxidation process impacts different constituents, including proteins, carbohydrates, lipids, pigments, vitamins, and DNA. The rate of oxidation rises with time, reducing the shelf-life of meat and meat products [65]. In particular, rabbit meat contains a high content of polyunsaturated fatty acids (the ratio of omega-6/omega-3 = 5.9) that provides a nutritional benefit. However, it also renders the meat more susceptible to lipid oxidation, affecting meat appearance and flavor [66]. In addition, lipid oxidation reduces the healthfulness of meat by causing the development of toxic compounds such as MDA and cholesterol oxidation products, which are harmful to human health [67,68]. The oxidative stability of rabbit meat can be improved by the dietary supplementation with natural antioxidants [2,35].

In addition, the current findings demonstrate that supplementation with GEO at a high dose improved the redox status balance in plasma, by increasing the TAC level by 11.64% and decreasing the MDA level by 7.60% compared to the control. In the same vein, the inclusion of ginger powder and oils in the diet of broiler chickens did not affect blood parameters, but they increased serum TAC levels and lowered MDA more than those of the control group [69].

## 5. Conclusions

In conclusion, the dietary supplementation of rosemary and ginger essential oils, especially at a high dose (0.5%), induced an improvement in the growth performance, feed utilization, and meat quality of the growing NZW rabbits. Body weight gain and feed conversion ratio significantly improved with both rosemary and ginger essential oils supplementation. The levels of cholesterol in muscle and plasma, as well as triglycerides in plasma, were significantly reduced. In addition, muscle fat content tended to decrease in the muscles of rabbits treated with the high level of both essential oils. In addition, rosemary and ginger essential oils attenuated the oxidant and antioxidant balance in the treated animals. This improvement may be reflected positively on rabbit production towards high quality, healthy meat, and sustainable production.

## Figures and Tables

**Table 1:** The ingredients and calculated chemical composition of the experimental diet.

Ingredients	(g/kg)	Calculated Chemical Composition	(g/kg as Fed Basis)
Lucerne hay	365	Crude protein (CP)	177.2
Ground barley grains	160	Ether extract (EE)	24.6
Ground yellow corn	120	Crude fiber (CF)	127.6
Wheat barn	180	Calcium (Ca)	10.6
Soybean meal (44% CP)	130	Total phosphorus (P)	3.8
Common Salt (NaCl)	5	Lysine	9.1
Beet molasses	20	Methionine	4.3
Dicalcium phosphate	5	Methionine + Cystine	7.7
Ground limestone	10	Digestible energy (DE), kcal/kg	2574
Vit. & Min. Premix ^1^	2		
DL-Methionine	2		
Anti-toxicants	1		

^1^ Each kg contains: Vit. A, 20,000 IU; Vit. E, 8.33 g; Vit. D_3_, 15,000 IU; Vit. K, 0.33 g; Vit. B_1_, 1.0 g; Vit. B_2_, 1.0 g; Vit. B_6_, 0.33 g; Vit. B_12_, 1.7 mg; Vit. B_5_, 8.33 g; Pantothenic acid, 3.33 g; Niacin, 8.33 g; Folic acid, 0.83 g; Biotin, 33 mg; Choline chloride, 20 g; Zn, 11.7 g; Fe, 12.5 g; Cu, 0.5 g; Co, 1.33 mg; Se, 16.6 mg; Mn, 5 g and antioxidants, 10 g.

**Table 2:** List of the active components profile of rosemary and ginger essential oils.

Component Identified	Area (%)
Rosemary Essential Oil (REO, *Rosmarinus officinalis*)	
Limonene	23.03
Cis- Vaccenic acid	12.91
Trans-4- Decadienal	10.67
Octane, 2, 4, 6- trimethyl	9.14
9, 12 Octadecadienoic acid	8.77
Trans-3-Nonene	7.65
4-Heptenal	6.9
Eucalyptol	3.98
Linalool	3.5
2-Decenal	2.96
2-Undecenal	2.49
Octadecanoic acid	1.61
Unidentified peaks	6.39
Ginger essential oil (GEO, *Zingiber Officinalis*)	
Zingiberene	29.74
Carveol	15.05
Cyclohexene.3-(1, 5-dimethyl-4-hexenyl)-6-methylene	10.4
9, 12 Octadecadienoic acid	9.2
Cis-alpha- Bisabolene	5.04
*n*-Hexadecanoic acid	3.93
Alpha- Farnesene	2.88
Unidentified peaks	23.76

**Table 3:** Effect of the dietary supplementation of rosemary and ginger essential oils on the growth performance of the growing NZW rabbits from 7th to 13th week of age (*n* = 24, 6 replicates in each group with 4 rabbits in each replicate).

Items	Control	REO-0.25	REO-0.5	GEO-0.25	GEO-0.5	SEM	*p*-Value
Initial body weight, g	853.4	850.1	849.5	848.4	853.1	2.16	0.543
Final body weight, g	1965 ^b^	1997 ^ab^	2068 ^a^	2000 ^ab^	2075 ^a^	20.22	0.001
Body weight gain, g	1112 ^b^	1147 ^b^	1218 ^a^	1152 ^b^	1222 ^a^	18.64	0.001
Average daily gain, g	22.69 ^b^	23.41 ^b^	24.86 ^a^	23.50 ^b^	24.93 ^a^	0.38	0.001
Daily feed intake, g	82.09 ^a^	81.25 ^ab^	80.21 ^b^	81.65 ^a^	79.85 ^b^	0.31	0.005
Feed conversion ratio, g	3.63 ^a^	3.48 ^ab^	3.22 ^b^	3.48 ^ab^	3.20 ^b^	0.05	0.001

^a–b^: Means in the same row bearing different superscripts are significantly different (*p* ≤ 0.05). REO-0.25 and REO-0.5: rosemary essential oil supplemented with 0.25 and 0.5%, respectively. GEO-0.25 and GEO-0.5: ginger essential oil supplemented with 0.25 and 0.5%, respectively. SEM: standard error of the mean.

**Table 4:** Effect of the dietary supplementation of rosemary and ginger essential oils on carcass traits of growing NZW rabbits at the end of the experiment (*n* = 6).

Items	Control	REO-0.25	REO-0.5	GEO-0.25	GEO-0.5	SEM	*p*-Value
Pre-slaughter live body weight, g	1978	2000	2065	2011	2060	6.67	0.812
Hot eviscerated carcass with a head, g	1185	1212	1254	1244	1242	51.5	0.728
Dressed carcass, %	59.89	60.57	60.72	61.82	60.29	1.19	0.575
Liver, %	2.22 ^c^	2.40 ^b^	2.46 ^ab^	2.55 ^a^	2.52 ^a^	0.04	<0.001
Kidneys, %	0.54	0.53	0.52	0.53	0.54	0.02	0.913
Heart, %	0.27	0.26	0.25	0.26	0.27	0.01	0.650
Spleen, %	0.06	0.05	0.06	0.05	0.06	0.01	0.908
Head, %	4.93	4.87	4.81	4.88	4.97	0.13	0.810
Giblets, %	3.02 ^c^	3.19 ^b^	3.23 ^ab^	3.34 ^a^	3.32 ^a^	0.03	<0.001
Total edible parts, %	62.91	63.75	64.69	65.16	65.26	0.45	0.439
Non-edible parts, %	37.09	36.25	35.31	34.84	34.74	0.45	0.439

^a–c^: Means in the same row bearing different superscripts are significantly different (*p* ≤ 0.05). REO-0.25 and REO-0.5: rosemary essential oil supplemented with 0.25 and 0.5%, respectively. GEO-0.25 and GEO-0.5: ginger essential oil supplemented with 0.25 and 0.5%, respectively. SEM: standard error of the mean.

**Table 5:** Effect of the dietary supplementation of rosemary and ginger essential oils on meat composition traits of the growing NZW rabbits at the end of the experiment (*n* = 6).

Items	Control	REO-0.25	REO-0.5	GEO-0.25	GEO-0.5	SEM	*p*-Value
Longissimus dorsi muscle							
Moisture (g/100 g meat)	73.97	74.04	73.79	74.03	73.82	0.28	0.857
Protein (g/100 g meat)	24.53	24.55	24.93	24.74	24.92	0.17	0.457
Fat (g/100 g meat)	0.44	0.39	0.40	0.42	0.39	0.03	0.454
Ash (g/100 g meat)	1.30	1.30	1.27	1.27	1.28	0.05	0.955
Cholesterol (mg/100 g meat)	50.51 ^a^	49.51 ^ab^	48.20 ^b^	49.50 ^ab^	48.68 ^b^	0.41	0.035
TBARS (mg MDA/kg meat)	0.20	0.19	0.18	0.20	0.17	0.01	0.145
Hind leg muscle							
Moisture (g/100 g meat)	74.15	74.11	74.22	74.30	74.30	0.05	0.209
Protein (g/100 g meat)	22.09	22.04	22.03	22.14	22.18	0.08	0.699
Fat (g/100 g meat)	2.44	2.41	2.40	2.39	2.39	0.06	0.883
Ash (g/100 g meat)	1.25	1.24	1.24	1.26	1.24	0.02	0.840
Cholesterol (mg/100 g of meat)	66.06 ^a^	65.50 ^ab^	64.39 ^bc^	64.64 ^bc^	63.48 ^c^	0.18	0.003

^a,b,c^: Means in the same row bearing different superscripts are significantly different (*p* ≤ 0.05). REO-0.25 and REO-0.5: rosemary essential oil supplemented with 0.25 and 0.5%, respectively. GEO-0.25 and GEO-0.5: ginger essential oil supplemented with 0.25 and 0.5%, respectively. TBARS: thiobarbituric acid reactive substances. MDA: malondialdehyde. SEM: standard error of the mean.

## Data Availability

All relevant data are within the paper, and they are available from the corresponding authors.
